# Transkingdom network analysis reveals the dominant role of ileal microbiota in host metabolism over colonic microbiota in diet-induced obesity

**DOI:** 10.1128/msystems.01199-25

**Published:** 2025-10-31

**Authors:** Peng Chen, Xiajialong Li, Shenji Yang, Huiying Liu, Zhipeng Li

**Affiliations:** 1 The Second Affiliated Hospital, Jiangxi Medical College, Nanchang University, Nanchang, China; 2 State Key Laboratory of Food Science and Resources, China-Canada Joint Lab of Food Science and Technology (Nanchang), Nanchang University, Nanchang, China; Istanbul Medipol University School of Medicine, Istanbul, Turkey

**Keywords:** microbiota, ileum, colon, metabolic disorder, transkingdom network analysis

## Abstract

Distinct gut habitats shape the spatial organization of resident microbes, with most studies focusing on the colon/feces and ileum as key sites for host-microbiota interactions. However, the relationship between microbial biogeography and host metabolism remains poorly understood. Here, we investigated the differential responses of ileal and colonic microbiota to a high-fat diet using two-way analysis of variance. Furthermore, we explored their respective contributions to diet-induced metabolic disorders through transkingdom network analysis (TkNA), a well-established causal-inference approach. A total of 39 taxa, including *Bifidobacterium*, *Bacteroides*, and *Limosilactobacillus*, were regulated by diet in an intestinal segment-specific manner (FDR <5%). TkNA integrating microbiota and liver transcriptome data revealed that the ileal microbial network was topologically closer to the liver transcriptomic network and its main subnetworks compared to the colonic network. This pattern was consistent when comparing the ileal and colonic networks’ proximity to the serum and liver metabolomic network. Finally, as anticipated, TkNA demonstrated stronger associations between the ileal microbiota and metabolic parameters compared to those of the colonic microbiota. In conclusion, our comprehensive analysis reveals that ileal microbiota plays a more significant role than colonic microbiota in mediating dietary effects on host metabolism, offering valuable insights for future studies in determining the optimal gut microbiota sampling location.

**IMPORTANCE:**

Evidence shows that gut microbiota plays a crucial role in host metabolism, but its composition varies across intestinal segments. While the ileum is essential for nutrient absorption, the colon hosts a denser, more diverse microbiota involved in fiber fermentation. It remains unclear which segment has a greater impact on host metabolism. In this study, we used an advanced network analysis to integrate multi-omics data and perform causal inference. Compared to colonic microbiota, ileal microbiota showed stronger associations with liver gene expression, liver metabolites, serum metabolites, and host metabolic phenotypes. These findings suggest that the ileal microbiota has a more significant influence on host metabolism, highlighting its importance in future microbiota research.

## OBSERVATION

Microbial composition varies significantly across different intestinal segments, and the distinct contributions of these microbial communities to host metabolism remain poorly understood (1). The ileum is a critical site for food digestion and nutrient absorption (2, 3), while the colon harbors a higher microbial load and serves as a major site for dietary fiber fermentation (4). While fecal/colonic samples are often used due to their non-invasive accessibility (5), there is currently insufficient evidence to determine whether ileal or colonic microbiota should be prioritized in metabolic disorder-focused investigations.

Due to the interconnected nature of gut regions, definitively attributing metabolic effects to the microbiota of a specific segment remains challenging (6). Transkingdom Network Analysis (TkNA) is a well-established causal-inference framework that provides a comprehensive view of biological systems by integrating multi-omics (7). In this study, we used TkNA to identify the intestinal region whose microbiota better reflects host metabolic states in diet-induced obesity (DIO).

### Animals and diet

Twenty-four 5-week-old male C57BL/6 J mice were fed normal chow or a high-fat diet (HFD, 60% calories from fat) *ad libitum* for 9 weeks (*n* = 12). The study was approved by the Animal Ethics Committee of Nanchang University (Approval No.: NCULAE-20221030024) and followed NIH guidelines for laboratory animal care. Various metabolic parameters, including adiposity, glucose/insulin tolerance, blood lipids, etc., were determined according to methods detailed in the supplementary methods.

### Multi-omics analysis

Ileal and colonic contents were used for 16S rRNA gene sequencing, the liver for RNA-seq, and both serum and liver for untargeted metabolomics. Details of the methods are provided in the supplementary methods.

### Transkingdom network analysis

Transkingdom networks integrating multi-omics data were constructed according to previous research (8, 9). A detailed description of the methods is provided in the supplementary methods.

### Ileal and colonic microbes exhibit differential responses to dietary interventions

Under standard feeding conditions, ileal and colonic microbial communities differed significantly, with colonic microbiota showing higher diversity than the ileum (Table S1; Fig. S1). Notably, a total of 21 genera (e.g., *Faecalibacterium* and *Fusobacterium*) were specific to the ileum, while 30 genera (e.g., *Acidibacter* and *Romboutsia*) were unique to the colon (Fig. S1E). After HFD feeding, the microbiota in the ileum and colon exhibited distinct responses (Fig. S2; Table S2). Centered log-ratio transformation was applied to normalize the microbial data, followed by two-way ANOVA. This analysis identified 32 taxa with significantly different responses to diet between the ileum and colon (FDR <5%, Fig. S3; Table S2).

### The ileal microbiota exerts a more profound influence on hepatic metabolism than the colonic microbiota

**Fig 1:**
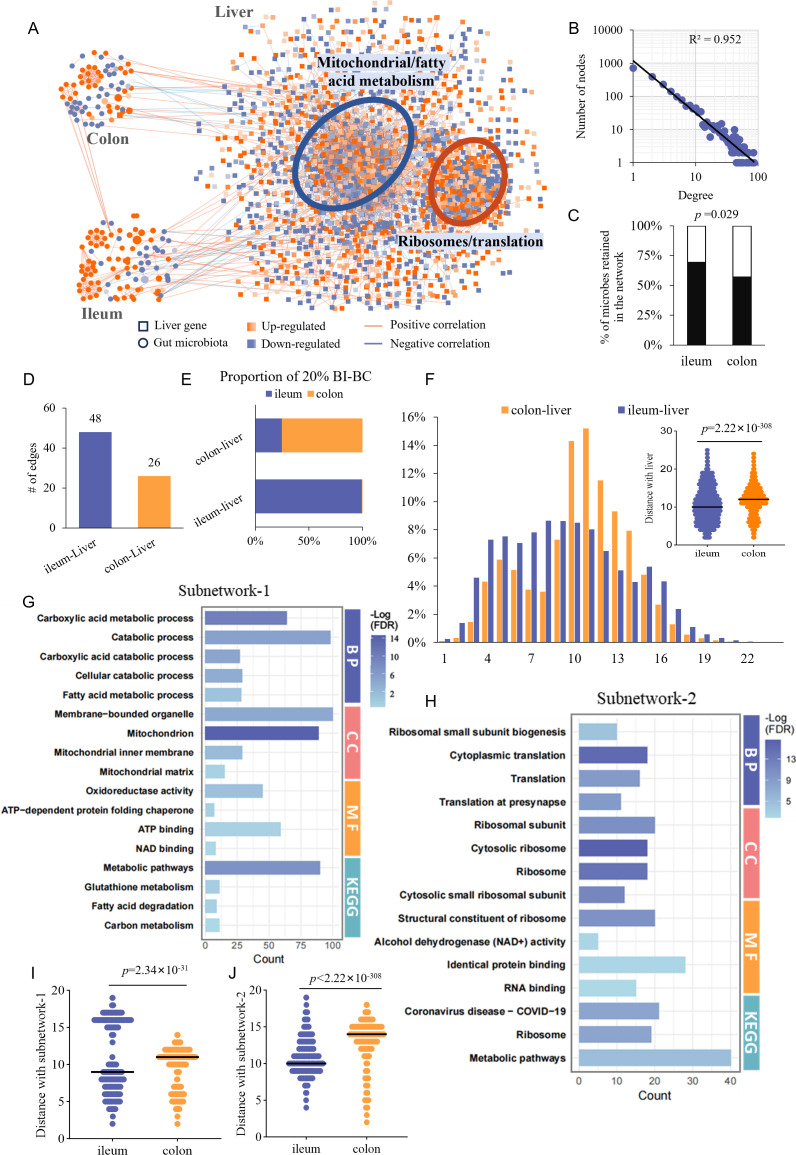
TkNA reveals closer connections between ileal microbiota and liver transcriptome compared to colonic microbiota. (**A**) Transkingdom network integrating the ileal/colonic microbiota and transcriptomic data. Each node represents a microbe or a gene, and each edge indicates a significant association between two nodes. Node color corresponds to the log fold change (HFD vs. NC) of the respective microbe or gene. (**B**) Distribution of node degree in the network. (**C**) Proportion of microbes retained in the network. A χ test was conducted to assess the difference. (**D**) Number of edges linking the ileum/colon to the liver. (**E**) To determine the likelihood of ileal/colonic microbes to be the hubs of the information between the ileal/colonic network and the liver, the BIBC of each microbial node was calculated. The proportion of microbes from the ileum/colon in the top 20% BIBC between that each microbial network and the transcriptome is shown, (**F**) Distribution of shortest path lengths between the nodes of the ileum/colon and transcriptome (the average shortest path is a metric evaluating closeness of the ileum/colon to the liver), (**G–H**) Gene enrichment analysis using Gene Ontology for the genes in subnetworks 1 and 2 in the liver (FDR <5%), (**I–J**) Distance of ileum/colon to subnetwork 1/2 (calculated similarly to panel F). The Wilcoxon test was used to test the difference between groups in panels **F**, **I**, and **J**.

We performed TkNA integrating microbiota and hepatic transcriptome data (Fig. 1A). The network included 98 ileal microbes, 78 colonic microbes, 2,059 liver genes, and 7,718 edges (Table S3), with a node degree distribution following the power law typical of regulatory biological networks (Fig. 1B). We analyzed network topology reflective of information flow in the network (9). The proportion of ileal microbes retained in the network was significantly higher than colonic microbes (Fig. 1C). More connections were observed between the ileum and liver than between the colon and liver (Fig. 1D). Notably, key nodes mediating ileum-liver information flow were exclusively ileal microbes, while colon-liver flow partly involved ileal microbes as well (Fig. 1E). Moreover, the distance between the ileum and liver (average length of all shortest paths) was significantly shorter than that between the colon and liver (Fig. 1F).

Two high-density sub-networks were identified in the transcriptomic network (Fig. 1A). Gene enrichment analysis indicated that the first sub-network was enriched in pathways related to fatty acid catabolism/mitochondria, while the second was associated with ribosome/protein translation (Fig. 1G and H; Table S4). The ileal microbiota was also closer to both sub-networks than the colonic microbiota (Fig. 1I and J). These findings suggest that ileal microbiota play a dominant role in HFD-induced liver metabolic changes, with a closer intrinsic link to liver metabolism than colonic microbiota. We have provided the BIBC values of microbes associated with the liver and its subnetworks (Table S5; Fig. S4). Several well-known genera (e.g., *Prevotella*, *Bacteroides*, and *Roseburia*) rank among the top ileal microbes mediating ileum-liver cross-talk.

### Ileal microbiota shows a stronger association with host metabolome and phenotypes than colonic microbiota

**Fig 2:**
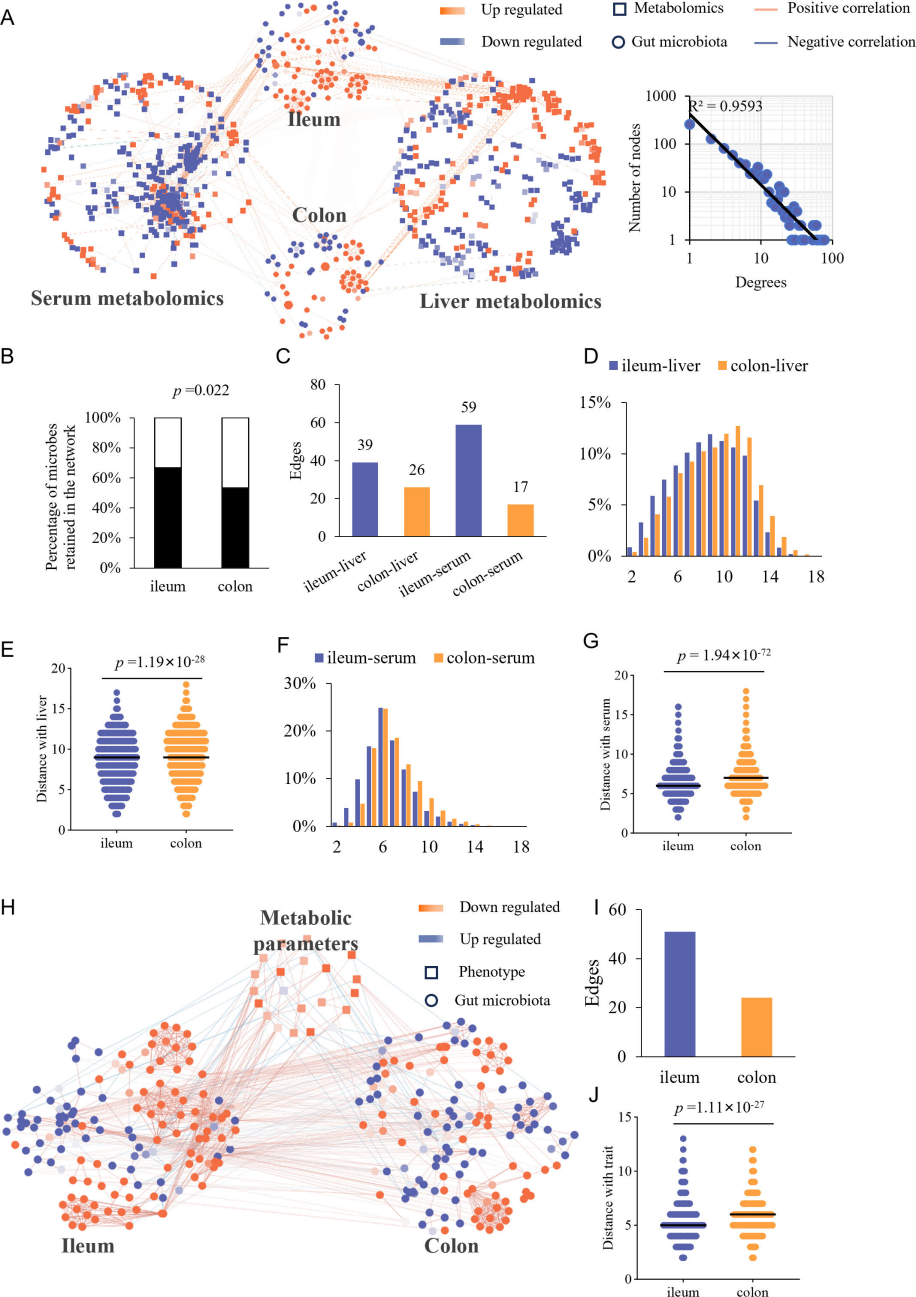
TkNA reveals that ileal microbiota is topologically closer to the host metabolome and phenotypic outcomes in DIO. (**A**) Transkingdom network integrating the microbiota and metabolomic data. Each node represents a microbe or a trait, and each edge indicates a significant association between two nodes. Node color corresponds to the log fold change (HFD vs. NC) of the respective nodes. (**B**) Proportion of microbes retained in the network (χ test was conducted to assess the difference), (**C**) Number of edges linking ileum/colon to serum/liver metabolome, (**D**) Distribution of shortest path lengths between the nodes of ileum/colon and liver metabolome, (**E**) Average shortest path as a metric evaluating closeness of ileum/colon to liver, (**F**) Distribution of shortest path lengths between the nodes of ileum/colon and serum metabolome, (**G**) Average shortest path between ileum/colon and serum metabolome, (**H**) Transkingdom network integrating the microbiota and phenotypic outcomes, (**I**) Number of edges linking ileum/colon to phenotypes, (**J**) Average shortest path between ileum/colon and phenotypes. The Wilcoxon test was used to test the difference in panel **E**, **G**, and **J**.

To determine whether this phenomenon extends to the metabolome, we analyzed serum and liver metabolites (Table S6) and built a network linking them to gut microbiota (Fig. 2A), comprising 94 ileal microbes, 73 colonic microbes, 355 serum metabolites, 338 liver metabolites, and 2,789 edges (Table S7).

Similarly, ileal microbes were more retained in the network than those in the colon (Fig. 2B). The ileal microbiota also exerted more connections with both liver and serum metabolomes (Fig. 2C). Furthermore, shortest-path analyzes revealed that the ileal microbiota was topologically closer to both the liver and serum metabolome than the colonic microbiota (Fig. 2D through G). We also predicted the importance of microbes in mediating information flow from the ileal/colonic microbiota to the serum/liver metabolome (Table S8; Fig. S4).

Next, we determined the metabolic parameters of mice, including body weight gain, adiposity, liver index, glucose/insulin tolerance, blood lipids (Fig. S5), and constructed a network integrating gut microbiota with these phenotypes (Table S9; Fig. 2H). The ileal microbiota had twice as many edges with phenotypes as the colonic microbiota (Fig. 2I), and topological analysis confirmed the ileal network’s closer connection to the phenotypic network (Fig. 2J; Fig. S4; Table S10).

The small and large intestines differ markedly in their anatomical structure and physiological environment, which contribute to the heterogeneity of their microbial communities (10). Hence, the microbiota in the ileum and colon may exhibit significant differences in their responses to diet and their influence on host metabolism. We compared the impact of ileal and colonic microbiota on metabolism using TkNA from the perspectives of transcriptomics, metabolomics, and phenotypic outcomes and found that ileal microbiota has a stronger influence on host metabolism.

The liver plays a central role in glucose/lipid metabolism (11). Lipid metabolism disorders and mitochondrial dysfunction in the liver are frequently reported in DIO (12), while HFD-induced rough endoplasmic reticulum stress disrupts ribosomal function and impairs protein synthesis (13, 14). Consistently, our transcriptomic network analysis revealed that HFD primarily affected two subnetworks: one associated with mitochondria/fatty acid metabolism and the other with ribosome/protein translation. Topological analysis showed that the ileal microbiota was more closely linked to the liver network and its subnetworks than the colonic microbiota.

The release of small molecules into circulation represents a key mechanism by which gut microbes regulate host metabolism (15). Most microbial metabolites are absorbed in the gut and transported to the liver via the portal vein (16). We found that ileal microbiota had a greater impact on serum and liver metabolites and phenotypic outcomes compared to colonic microbiota.

Despite its lower microbial density compared to the colon, the ileum is key for nutrient absorption and enterohepatic circulation (17). Furthermore, Peyer’s patches, the vagus nerve, and enteroendocrine cells primarily act in the small intestine (18, 19), implying the ileal microbiota’s stronger ties to immune, neural, and endocrine systems than the colonic microbiota. Consistently, our analysis offers data-driven evidence that substantiates this hypothesis.

In this study, we chose to analyze networks in a segmented manner rather than fully integrating all omic layers, as a significant imbalance in feature numbers and connectivity across layers would have reduced sensitivity and obscured segment-specific biological signals. While this approach preserves interpretability and biological relevance, it may limit the detection of cross-compartment interactions. Moreover, the meta-analytic approach in TkNA may potentially overlook biologically meaningful associations that are specific to either the HFD or NC condition. Thus, some context-dependent regulatory relationships may remain undetected, which is an inherent constraint of meta-analytic network construction. Additionally, the networks cannot capture all relevant biological variables, and the predicted results require validation through wet-lab experiments such as segment-specific microbiota manipulations. Nevertheless, this study provides a novel perspective on the differential impacts of ileal and colonic microbiota on host health, warranting further investigation in future research.

In conclusion, our analysis reveals that the ileal microbiota have a greater impact on host metabolism than colonic microbiota in DIO, offering insights for future studies in determining the optimal gut microbiota sampling location.

## Data Availability

The sequence data for gut microbiota and liver transcriptome that support the findings of this study are deposited at http://www.ncbi.nlm.nih.gov/bioproject/1260774 and https://www.ncbi.nlm.nih.gov/bioproject/1261153 respectively. Integrated sample mapping table linking each mouse/sample to all associated microbiome, metabolome, transcriptome, and phenotype data are provided in Table S11.

## SUPPLEMENTAL MATERIAL

Supplemental Figures msystems.01199-25-s0001.pdf Fig. S1-S5.

Supplemental Text msystems.01199-25-s0002.docx Supplemental methods.

Supplemental Tables msystems.01199-25-s0003.xlsx Tables S1-S11.

ASM does not own the copyrights to Supplemental Material that may be linked to, or accessed through, an article. The authors have granted ASM a non-exclusive, world-wide license to publish the Supplemental Material files. Please contact the corresponding author directly for reuse.
