# Supplementary material for: Transkingdom network analysis reveals the dominant role of ileal microbiota in host metabolism over colonic microbiota in diet-induced obesity
Source: mSystems. 2025 Oct 31;10(11):e01199-25. doi: 10.1128/msystems.01199-25 (PMC12625735; doi:10.1128/msystems.01199-25)
Supplement: Supplemental Figures — Fig. S1-S5. [file msystems.01199-25-s0001.pdf]

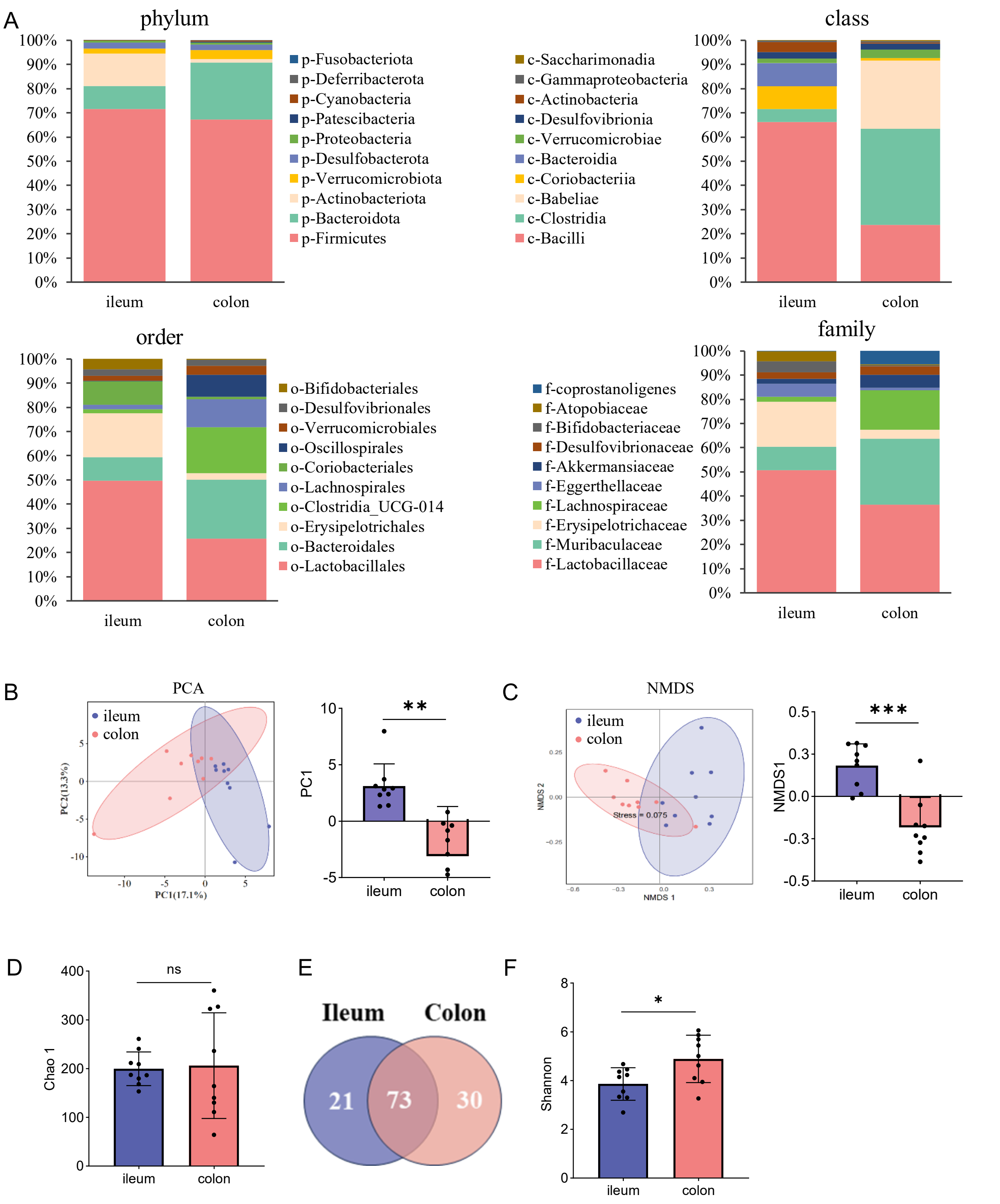

Fig. S1 The structure of the microbiota in the ileum and colon differs significantly. (**A**) Stacked bar charts showing to top taxa in ileum and colon at phylum, class, order, and family levels, (**B**) Principal component analysis, (**C**) Non-metric multidimensional scaling analysis, (**D**) Chao1 index, (**E**) Venn diagram at genus level, (**F**) Shannon index. Two-tailed t test was used to test difference between the two groups. n=9-10, *p<0.05, **p<0.01, ***p<0.001, ****p<0.001.

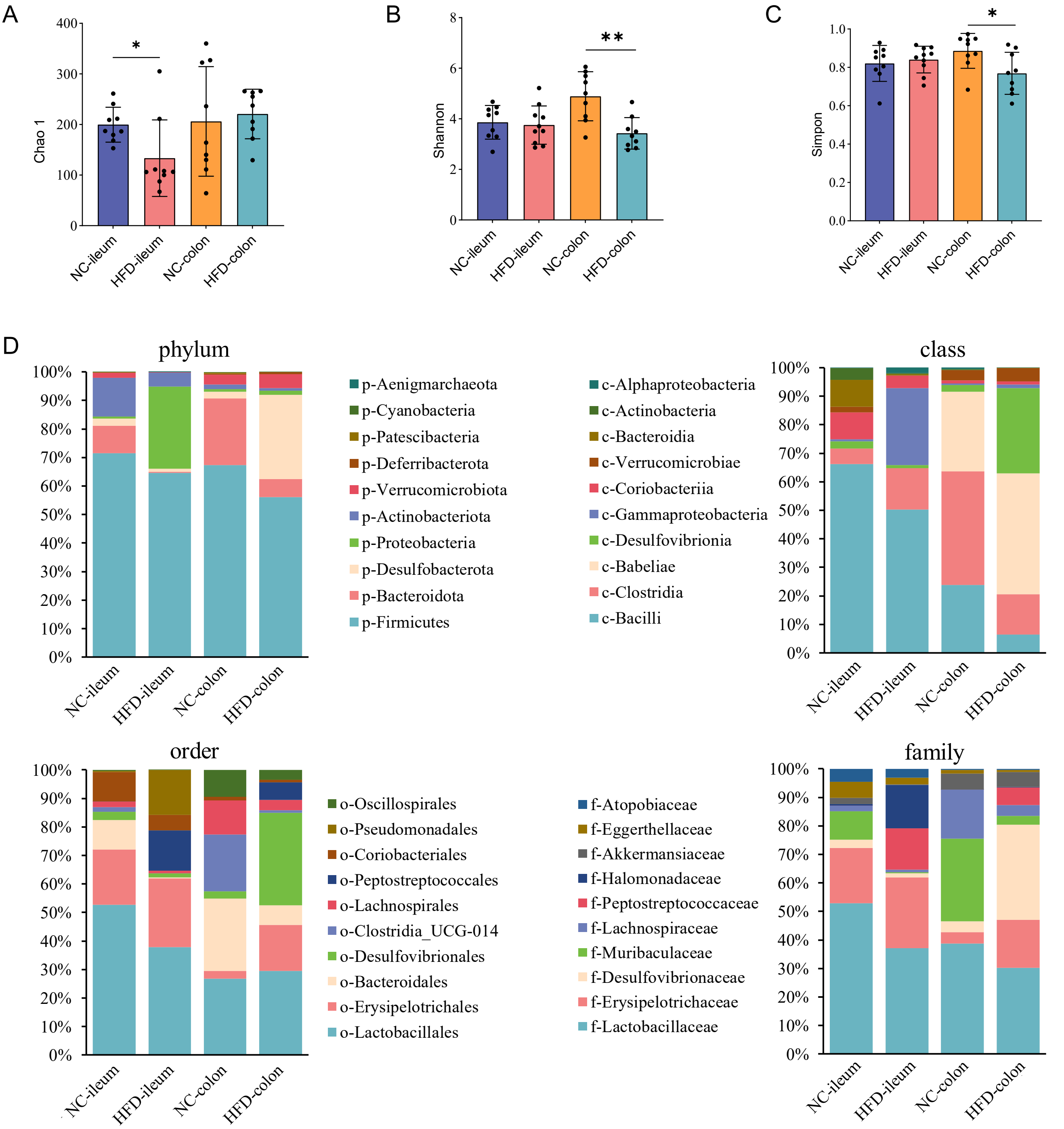

Fig. S2 Changes in the gut microbiota of the ileum and colon induced by a high-fat diet in mice. Mice were fed either a normal chow diet (NC) or a high-fat diet (HFD) for 9 weeks. The composition of gut microbiota in the ileal and colonic contents was analyzed using 16S rRNA gene sequencing. (**A**) Chao1 index, (**B**) Shannon index, (**C**) Simpson index, (**D**) Stacked bar charts showing to top taxa in ileum and colon at phylum, class, order, and family levels. Two-tailed t test was used to test difference between the two groups. n=9-10, **p*<0.05, ***p*<0.01.

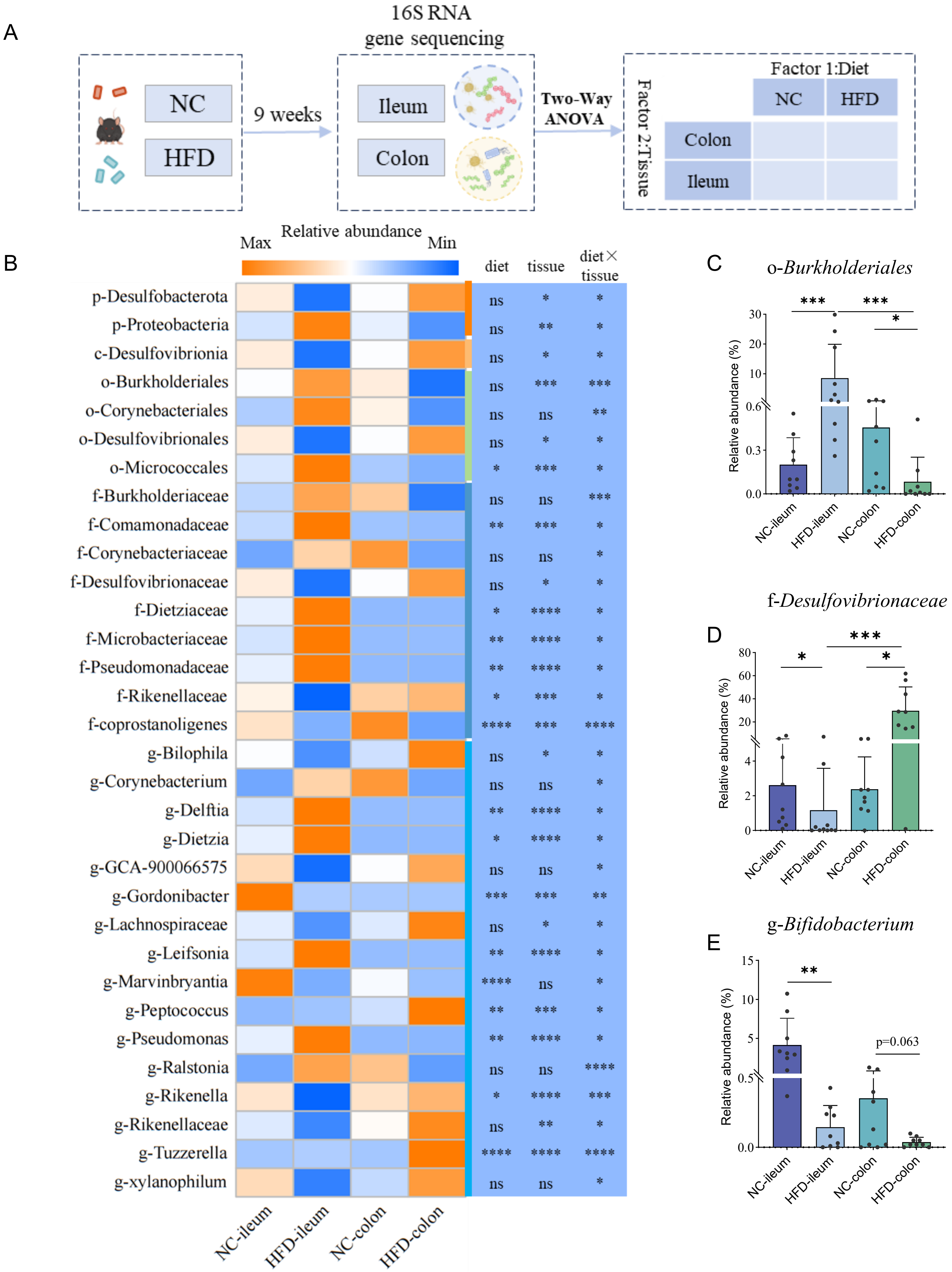

Fig. S3 A high-fat diet induced distinct changes in microbial abundance in the ileum and colon. (**A**) Experimental design. (**B**) A heatmap showing taxa that were significantly regulated by the interaction effect of diet (HFD/NC) and tissue (ileum/colon) analyzed via two-way ANOVA. (**C**) Burkholderiales was enriched by HFD in ileum, but decreased by HFD in colon. (**D**) *Desulfovibrionaceae* was not changed by HFD in ileum, but significantly enriched by HFD in colon. (**E**) *Bifidobacterium* was downregulated 28-fold in the ileum, whereas it was only downregulated 9-fold in the colon. n=9-10, **FDR*<0.05, ***FDR*<0.01, *** *FDR* <0.001, **** *FDR* <0.001. NC, normal chow diet.

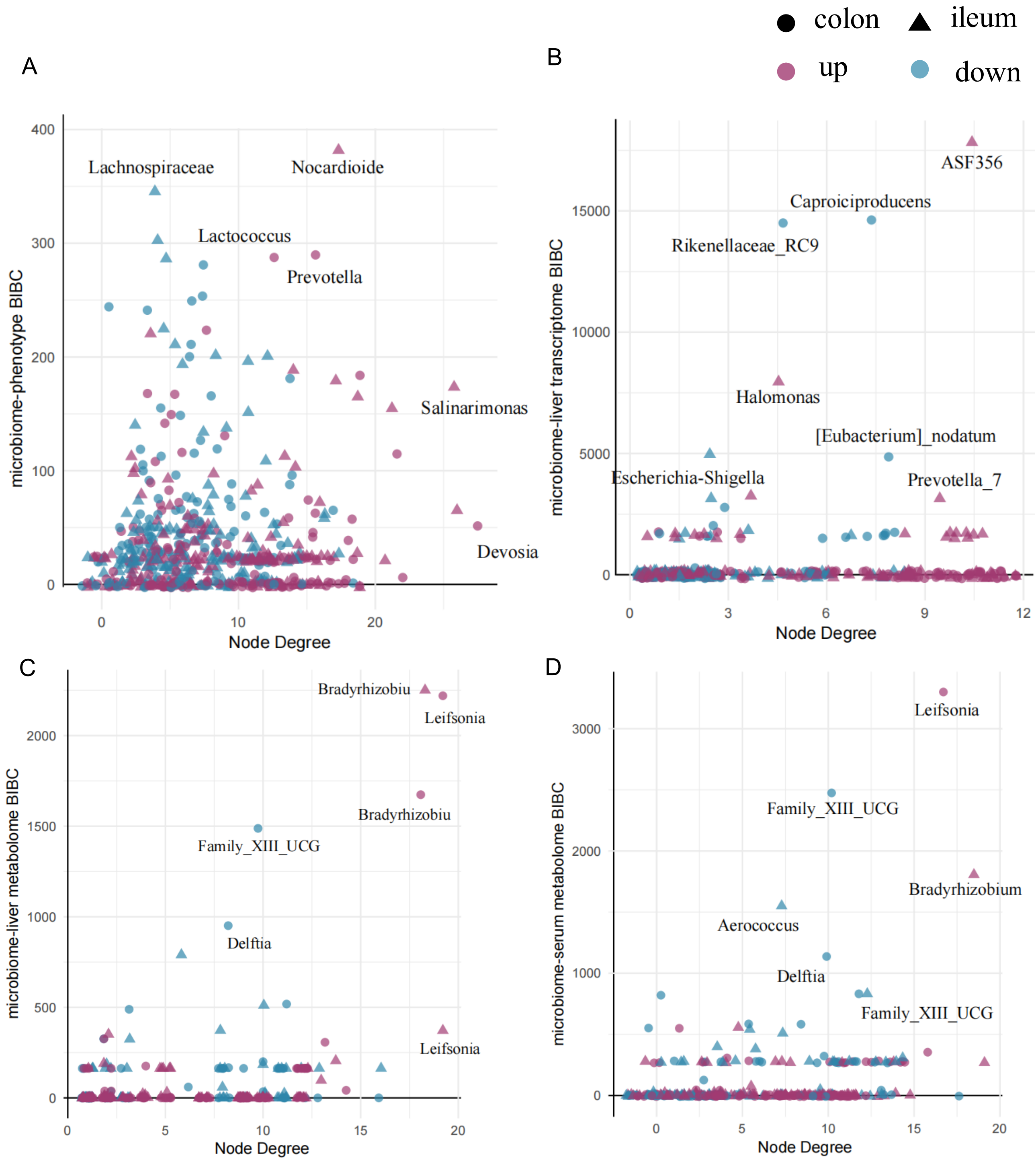

Fig. S4. Topological importance of ileal and colonic bacteria in multi-omics networks. The X-axis represents the degree of each bacterium in the network, while the Y-axis indicates the BIBC value of each bacterium between the gut microbiota and the corresponding omics network. (**A**) Degree and BIBC of each microbe in the gut microbiota-phenotype network. (**B**) Degree and BIBC of each microbe in the gut microbiota-hepatic transcriptome network. (**C**) Degree and BIBC of each microbe in the gut microbiota-hepatic metabolome network. (**D**) Degree and BIBC of each microbe in the gut microbiota-serum metabolome network.

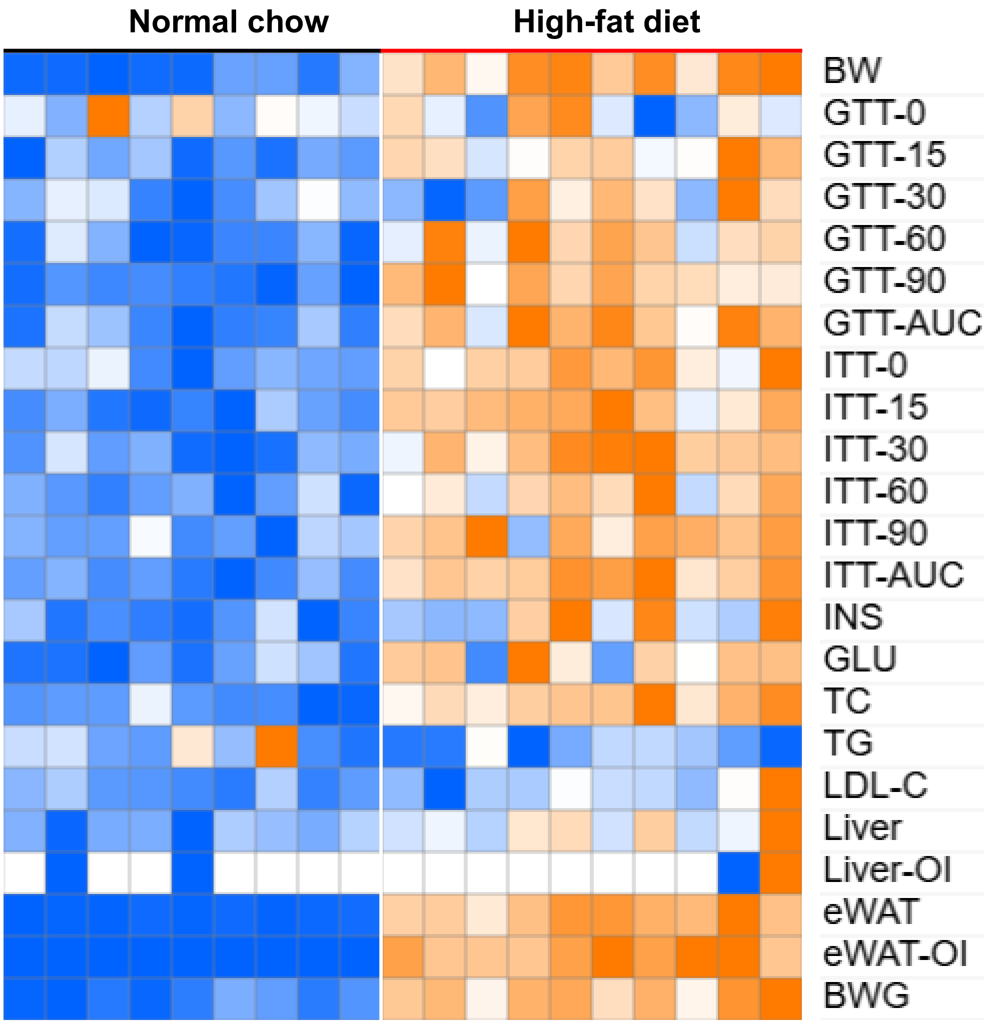

Fig. S5 Influence of high-fat diet on various metabolic parameters in mice. Mice were fed either a normal chow diet (NC) or a high-fat diet (HFD) for 9 weeks. BW, body weight; GTT-0, GTT-15, GTT-30, GTT-60, GTT-90, levels of blood glucose in glucose tolerance test at different time points; AUC, area under curve; ITT-0, ITT-15, ITT-30, ITT-60, ITT-90, levels of blood glucose in insulin tolerance test at different time points; INS, insulin levels; TC, total cholesterol; TG, total triglycerides; LDL-C, low-density lipoprotein cholesterol; Liver, liver weight; OI, organ index; eWAT, epididymal adipose tissue, BWG, body weight gain.
