# Supplementary material for: Transkingdom network analysis reveals the dominant role of ileal microbiota in host metabolism over colonic microbiota in diet-induced obesity
Source: mSystems. 2025 Oct 31;10(11):e01199-25. doi: 10.1128/msystems.01199-25 (PMC12625735; doi:10.1128/msystems.01199-25)
Supplement: Supplemental Text — Supplemental methods. [file msystems.01199-25-s0002.docx]

**Supplementary methods**

**1. Glucose and insulin tolerance test**

Glucose tolerance tests (GTT) were performed on mice fasted for 6 h and administered oral glucose (2 g/kg). Blood glucose was measured via tail vein using a glucometer (Accu Check, Roche) at 0, 15-, 30-, 60-, and 90-min post-administration. For the insulin tolerance test (ITT), mice fasted for 6 h received insulin (i.p. 1 U/kg). Blood glucose was measured at the same time points as in the GTT.

**2. Lipid profile analysis**

The levels of TC, TG, LDL-C, and HDL-C in mouse serum were measured using assay kits (Nanjing Jiancheng Bioengineering Institute), following the manufacturer's instructions.

**3. 16S RNA gene sequencing**

The detailed experimental procedures were adapted from a previous study [1]. Briefly, genomic DNA was isolated from ileal and colonic contents using the FastPure Stool DNA Isolation Kit (MJYH, China). The V3-V4 hypervariable regions of the 16S rRNA gene were amplified and sequenced on the Illumina NextSeq 2000 platform following established methodologies. Sequencing libraries were constructed with the NEXTFLEX Rapid DNA-Seq Kit (Bioo Scientific, USA) through PCR enrichment and adapter ligation.

**4. RNA-seq**

Total RNA was extracted from mouse liver using TRIzol® Reagent (Invitrogen, USA) according to the manufacture’s protocols. RNA sequencing was conducted following a protocol described in previous research [2]. Briefly, mRNA was purified, and sequencing libraries were constructed using NovoMagic (Beijing, China). Sequencing libraries were prepared from high-quality RNA (OD260/280: 1.8-2.2; RQN>6.5) through poly(A) selection, mRNA fragmentation (~300 bp), and double-stranded cDNA synthesis with adapter ligation. Libraries were sequenced on the Illumina NovaSeq X Plus platform. DESeq2 was employed to normalize the raw count data and perform differential expression analysis.

**5. Untargeted metabolomics**

Untargeted metabolomics analysis of serum and liver tissues was performed using liquid chromatography (Waters HSS T3 C18 column) coupled with an AB SCIEX TripleTOF 5600+ mass spectrometry system in information-dependent acquisition (IDA) mode. Metabolite extraction (acetonitrile precipitation), chromatographic gradients, and mass calibration followed established protocols [3]. Metabolite identification was carried out using the MENTLIN database normalization method.

**6. Two-Way Analysis of Variance (ANOVA)**

To assess the differential effects of diet on microbial abundance in the ileum and colon, a two-way ANOVA was conducted with diet (HFD/NC) as one factor, tissue (ileum/colon) as the second factor, and their interaction (diet × tissue) included in the analysis. If the interaction effect was significant (FDR<5%), the effect of diet on microbial abundance was considered tissue-specific.

**7. Transkingdom Network analysis**

**Network construction** Ileal/colonic microbiota and genes significantly regulated by HFD/NC in the liver (FDR<15%) were used as nodes to construct microbiota-transcriptome network. Spearman rank correlations were calculated between all pairs of nodes in each group (HFD and NC) of the experiment. A combined Fisher’s p value was calculated for each pair from the correlation p values from eachgroup. An FDR was calculated on the combined p values. Edges satisfied the following criteria were retained: (i) the sign of correlation coefficients were consistent in the two groups; (ii), the final FDR for the correlation is less than 15%; (iii) the edges satisfied principles of causality [4] (i.e., for any pair of nodes, the fold changes in the HFD vs. NC comparison is concordant with the sign of the correlation between these two nodes). The network was visualized in Cytoscape.

Similarly, ileal/colonic microbiota and metabolites that were significantly regulated by HFD/NC in the serum and liver (*p*<0.05) were used as nodes to construct the microbiota-metabolome network. Ileal/colonic microbiota and various metabolic parameters regulated by HFD were used as nodes to construct the microbiota-phenotype network.

**Detecting subnetworks** To identify subnetworks of correlated genes in liver, we used the MCODE (Molecular Complex Detection) plug-in for Cytoscape to identify clusters (subnetworks) of correlated genes. The largest two subnetworks in liver were selected for further analyses. Gene enrichment analysis all performed on the list of genes in each subnetwork.

**Shortest paths analysis** All shortest paths between ileal/colonic microbiota and the liver transcriptome, serum metabolome, liver metabolome, and phenotypic outcomes were analyzed using the R package igraph (version 2.1.1). The median length of the shortest paths was used to evaluate the distance between ileal/colonic microbiota and other networks, with Wilcoxon rank-sum test used to test the statistical significance.

**Identifying important nodes for information flow** Bi-partite betweenness centrality (BIBC) has previously been used to find nodes that control the information flow between two parts of a network [5]. Node BIBC was calculated between ileal/colonic microbiota and the liver network. we counted the number of microbial nodes in the top 20% (ranked in decreasing order of BIBC) belonging to ileal or colonic microbes.

[1] L. Wang, Z. Deng, Y. Li, Y. Wu, R. Yao, Y. Cao, M. Wang, F. Zhou, H. Zhu, H. Kang, Ameliorative effects of mesenchymal stromal cells on senescence associated phenotypes in naturally aged rats, Journal of translational medicine. 22 (2024) 722.

[2] W. Huang, W. Zhu, Y. Lin, F.K. Chan, Z. Xu, S.C. Ng, Roseburia hominis improves host metabolism in diet-induced obesity, Gut Microbes. 17 (2025) 2467193.

[3] Q. Nie, M. Xing, H. Chen, J. Hu, S. Nie, Metabolomics and lipidomics profiling reveals hypocholesterolemic and hypolipidemic effects of arabinoxylan on type 2 diabetic rats, Journal of agricultural and food chemistry. 67 (2019) 10614-10623.

[4] A. Yambartsev, M.A. Perlin, Y. Kovchegov, N. Shulzhenko, K.L. Mine, X. Dong, A. Morgun, Unexpected links reflect the noise in networks, Biol Direct. 11 (2016) 52.

[5] A. Morgun, A. Dzutsev, X. Dong, R.L. Greer, D.J. Sexton, J. Ravel, M. Schuster, W. Hsiao, P. Matzinger, N. Shulzhenko, Uncovering effects of antibiotics on the host and microbiota using transkingdom gene networks, Gut. 64 (2015) 1732-1743.
